# Effects of Moderate Amounts of Barley in Late Pregnancy on Growth, Glucose Metabolism and Osteoarticular Status of Pre-Weaning Horses

**DOI:** 10.1371/journal.pone.0122596

**Published:** 2015-04-13

**Authors:** Pauline Peugnet, Morgane Robles, Luis Mendoza, Laurence Wimel, Cédric Dubois, Michèle Dahirel, Daniel Guillaume, Sylvaine Camous, Valérie Berthelot, Marie-Pierre Toquet, Eric Richard, Charlotte Sandersen, Stéphane Chaffaux, Jean-Philippe Lejeune, Anne Tarrade, Didier Serteyn, Pascale Chavatte-Palmer

**Affiliations:** 1 INRA, UMR1198 Biologie du Développement et Reproduction, F-78350, Jouy en Josas, France; 2 IFCE, Station Expérimentale de la Valade, F-19370, Chamberet, France; 3 INRA, UMR85, Physiologie de la Reproduction et Comportements, CNRS, UMR7247, F-37380, Nouzilly, France, Université François Rabelais de Tours, F-37000, Tours, France; 4 AgroParis Tech, F-75005, Paris, France; 5 Normandie University, UNICAEN, SF4206 ICORE / LABEO Frank Duncombe Laboratory, F-14000, Caen, France; 6 Clinique Equine, Faculté de Médecine Vétérinaire, Université de Liège, B-4000, Liège, Belgium; Sonoma State University, UNITED STATES

## Abstract

In stud management, broodmares are commonly fed concentrates in late pregnancy. This practice, however, was shown to correlate with an increased incidence of osteochondrosis in foals, which may be related to insulin sensitivity. We hypothesized that supplementation of the mare with barley in the last trimester of pregnancy alters the pre-weaning foal growth, glucose metabolism and osteoarticular status. Here, pregnant multiparous saddlebred mares were fed forage only (group F, n=13) or both forage and cracked barley (group B, n=12) from the 7^th^ month of pregnancy until term, as calculated to cover nutritional needs of broodmares. Diets were given in two daily meals. All mares and foals returned to pasture after parturition. Post-natal growth, glucose metabolism and osteoarticular status were investigated in pre-weaning foals. B mares maintained an optimal body condition score (>3.5), whereas that of F mares decreased and remained low (<2.5) up to 3 months of lactation, with a significantly lower bodyweight (-7%) than B mares throughout the last 2 months of pregnancy. B mares had increased plasma glucose and insulin after the first meal and after the second meal to a lesser extent, which was not observed in F mares. B mares also had increased insulin secretion during an intravenous glucose tolerance test (IVGTT). Plasma NEFA and leptin were only temporarily affected by diet in mares during pregnancy or in early lactation. Neonatal B foals had increased serum osteocalcin and slightly increased glucose increments and clearance after glucose injection, but these effects had vanished at weaning. Body measurements, plasma IGF-1, T_4_, T_3_, NEFA and leptin concentrations, insulin secretion during IVGTT, as well as glucose metabolism rate during euglycemic hyperinsulinemic clamps after weaning, did not differ between groups. Radiographic examination of joints indicated increased osteochondrosis relative risk in B foals, but this was not significant. These data demonstrate that B or F maternal nutrition has very few effects on foal growth, endocrinology and glucose homeostasis until weaning, but may induce cartilage lesions.

## Introduction

Epidemiological observations in humans have linked early-life events with a range of degenerative pathologies in adulthood. Individuals with small birth weight are at greater risk of developing coronary heart disease, hypertension, type II diabetes or osteoporosis in later life [1]. Because fetal growth is primarily determined by the nutrient supply, studies of the Developmental Origins of Health and Disease have first focused on maternal undernutrition and nutritional deficiencies. Indeed, individuals who were exposed *in utero* to the Dutch Famine were more susceptible to develop metabolic syndrome in adult life [2]. The concept was extended to all forms of malnutrition, since higher risk to develop diseases as an adult was found in both individuals born small and individuals born large, with a U-shaped curve [3, 4].

Developmental programming of the offspring by maternal nutritional imbalance has been investigated experimentally in mammals using various models aimed at compromising the fetal nutrient supply [5]. In horses, there is a growing body of evidence indicating immediate and long term post-natal consequences of fetal adaptations to intra-uterine *stimuli*. Experimental models of lush *versus* deprived fetal nutrient supply have been obtained by transferring embryos from small breeds (ponies) into mares from large breeds (Thoroughbred, saddlebred and draft mares) and embryos from large breeds into mares from small breeds. Growth patterns were altered up to three years of age [6–8]. Glucose homeostasis, the cardiovascular function, as well as thyroid hormones concentrations, were affected until weaning [8–10].

The effect of maternal nutrition was partly investigated by Ousey and her colleagues: feeding pregnant mares with moderate *versus* high amounts of concentrates throughout pregnancy did not affect birth weight, but enhanced pancreatic beta-cell sensitivity to glucose in newborns born to mares in the moderate group [11]. Besides, feeding pregnant dams with a high starch diet from the 7^th^ month of pregnancy induced a trend towards lower insulin sensitivity in foals at 160 days of age [12]. Recently, an epidemiological study demonstrated that foals born to dams supplemented with concentrates during pregnancy had an increased incidence of osteochondrosis lesions compared to those born to dams that were not supplemented [13].

In practice, broodmares are often fed high energy rations in late pregnancy *i*.*e*., at the time of maximal fetal growth, which may have detrimental effects in the newborn but also in the adult horse. The present study investigates the effects of adding a limited amount of carbohydrates-concentrated feed to the diet of broodmares on their foals’ post-natal growth, glucose metabolism and joint health. Cracked barley was chosen as a concentrate in order to provide additional energy to mares for the last four months of pregnancy and because it is commonly used in stud management. Foals were monitored from birth to weaning for growth, glucose homeostasis, endocrine factors involved in both growth and energy regulation (Insulin-like Growth Factor-I, IGF-I; thyroid hormones, T_4_ and T_3_), as well as biomarkers of bone cell activity during growth (osteocalcin; hydroxyproline; C-telopeptide of type II collagen, CTX-II).

## Materials and Methods

### Animals

Animal studies received ethical approval from the local ethics committee (“Comité Régional d’Ethique pour l’Expérimentation Animale du Limousin”) under protocol number 5-2013-5.

#### Mares and establishment of pregnancies

Twenty-five mares (Selle Français, Anglo-Arab and Saddlebred) were used (median age: 9 years; range 6 to 21). They were raised in the “Institut Français du Cheval et de l’Equitation” experimental farm (Chamberet, France, 45°34'55.17''N, 1°43'16.29''E, 442 m). All mares were multiparous (median parity: 3; range 2 to 11 foals). Twenty-five pregnancies were obtained by artificial insemination (from May 9^th^ to June 29^th^) using semen from one saddlebred stallion.

#### Management and feeding of mares and foals

From ovulation, grazing was available 24 h/day with free access to water and mineral salts. Pregnant mares were managed in one same herd. From November 21^st^ (median gestational day 186; range 145 to 196), they were moved to individual boxes in the same barn. They were fed hay and hay-crop silage (haylage) until December 5^th^ (median gestational day 200, range 159 to 210), when they were allocated to a “forage” (group F, n = 13) or “barley” (group B, n = 12) feeding group, with equal repartition between groups according to age, parity and withers’ height (Table 1). Group F was fed hay and haylage, whereas group B was fed hay, haylage and a homemade mix of cracked barley with vitamins and minerals (Excel Prima S, Chauveau Nutrition, Cholet, France). Group F received vitamins and minerals simultaneously to concentrates distribution in group B. Diets were distributed in two daily meals (8:45 AM and 3:45 PM), with free access to water. Dietary offer was adjusted according to mares’ bodyweight at median gestational days 208, 221, 241, 272, 282, 304, 321 and 332, in accordance with current recommendations for broodmares [14]. A different hay batch was used near parturition because the batch used at the start of the experiment was out of stock. The quality of feedstuff was measured (Table 2). The daily quantity of feeds given to mares according to gestational age is shown in Table 3.

**Table 1:** Repartition of broodmares between groups “forage” and “barley” according to age, parity and withers’ height (median [quartile 1-quartile 3], minimum and maximum values).

	Group “forage” (n = 13)			Group “barley” (n = 12)		
	SF n = 6, AA n = 6, SD n = 1			SF n = 8, AA n = 3, SD n = 1		
	Median [Q1–Q3]	Min	Max	Median [quartile 1-quartile 3]	Min	Max
**Parity (number of foals)**	3 [2–3]	2	11	3 [2–4]	2	8
**Age (years)**	9.2 [8.1–10.2]	6.1	21.2	8.6 [6.9–12.8]	6.1	19.1
**Withers’ height (cm)**	162.0 [160.0–165.0]	157.0	170.0	162.5 [160.5–165.0]	156.0	168.0

SF: Selle Français, AA: Anglo-Arab or Anglo-Arabian type, SD: Saddlebred.

**Table 2:** Quality of feedstuff given to broodmares of groups “forage” and “barley” from November (median gestational day 186) to parturition.

	Chemical composition (per kg of dry matter)			Mineral composition (per kg of dry matter)	
	Net energy (Horse feed units)	Horse digestible crude proteins (g)	Raw cellulose (g)	Calcium (g)	Phosphorus (g)
**Homemade mix**	1.2	127	52.7	12.1	5.8
**Haylage**	0.6	104	249.0	7.7	3.8
**Hay H1**	0.5	30	372.0	3.7	2.1
**Hay H2**	0.6	88	30.8	6.6	3.8
**Excel Prima S**	NA	NA	NA	375	62.5

**Table 3:** Daily individual quantities of feeds given to pregnant mares of groups “forage” and “barley” from November (median gestational day 186) to parturition. H1 and H2 indicate when a different hay batch was used.

Median gestational day	Meal	Type of hay	Group “barley”			Group “forage”		
			Hay (kg)	Haylage (kg)	Barley mix (kg)	Hay (kg)	Haylage (kg)	Minerals and vitamins (kg)
**186**	**8:45 AM**	H1	3	1.5	0	3	1.5	0
	**3:45 PM**	H1	5	1.5	0	5	1.5	0
**200**	**8:45 AM**	H1	3	1	1	3	1.5	0.027
	**3:45 PM**	H1	5	1	1	6	1.5	0.028
**208**	**8:45 AM**	H1	3	1.5	1.5	3	1.5	0.027
	**3:45 PM**	H1	4	1.5	1.5	6	1.5	0.028
**221**	**8:45 AM**	H1	3	1.5	1.5	3	1.5	0.027
	**3:45 PM**	H1	5	2	1.5	6.5	2	0.028
**241**	**8:45 AM**	H1	3	2.5	1.5	3	1.5	0.027
	**3:45 PM**	H1	4	2.5	2	6.5	2	0.028
**272**	**8:45 AM**	H1	3	2.5	1.5	4	2	0.030
	**3:45 PM**	H1	4	2.5	2	8	2	0.030
**282**	**8:45 AM**	H1	1	3	1.5	3	2	0.030
	**3:45 PM**	H1	3	6	2	6.5	5.5	0.030
**304**	**8:45 AM**	H2	1	3	1.5	3	2	0.030
	**3:45 PM**	H2	3	6	2	6.5	5.5	0.030
**321**	**8:45 AM**	H2	1	3	1.5	3	3	0.035
	**3:45 PM**	H2	3	6	2	7	6	0.035
**332**	**8:45 AM**	H2	1	3	1.5	3	3	0.035
	**3:45 PM**	H2	3	6	2	7	6	0.035

All foals were born during spring (from April 8^th^ to June 3^rd^). Mares and foals returned to grazing 3 days after foaling. Fillies and colts were raised in one group in the same pasture until weaning at 180 days of age by complete, abrupt separation of mares and foals. Foals were then housed in open barns, fed hay twice a day and supplemented individually (using an automatic concentrates feeder) with homemade pellets containing barley, soybean cake, molasses and vitamins and minerals (Excel Prima S, Chauveau Nutrition, Cholet, France) in agreement with current recommendations for growing foals [14], with free access to water. The daily nutritional supply to weanlings is given in Table 4. Foals were dewormed at median age 142 days (range 105 to 161).

**Table 4:** Daily nutritional supply to weanlings (6 to 12 months of age).

	Chemical composition (per kg of dry matter)			Mineral composition (per kg of dry matter)	
	Net energy (Horse feed units)	Horse digestible crude proteins (g)	Raw cellulose (g)	Calcium (g)	Phosphorus (g)
**Homemade pellet**	1.13	102	63.9	7.10	4.90
**Hay**	0.62	95	315.5	5.85	2.38

All mares returned to farm without further experimentation after weaning, whereas foals were kept for further research beyond weaning.

#### Body measurements and blood sampling in mares and foals

All measurements and sampling were performed without analgesia or anesthesia.

Mares were weighed and their body condition score (BCS—scale 1 to 5 [15]) was estimated by two trained, independent operators monthly during pregnancy, in the 1^st^ day, the 1^st^, 2^nd^, 3^rd^ and 6^th^ month of lactation. Jugular vein blood samples were collected in EDTA tubes monthly during pregnancy, in the 1^st^ day, the 1^st^, 3^rd^ and 6^th^ month of lactation between 9:00 AM and 10:00 AM to measure plasma concentrations of non-esterified fatty acids (NEFA) which reflect the mobilization of body fat in case of energy deficit and leptin which are a proxy for adiposity.

Foals were weighed and measured for withers’ height, front leg length, chest circumference, shoulder and hip widths in the morning following birth and then monthly until weaning at 180 days of age. Jugular vein blood samples were collected in EDTA and serum tubes before first suckling, at 3 and 30 days of age after 4 h fasting, at 60 and 90 days of age after 6 h fasting and at 180 days of age after overnight fasting to measure plasma concentrations of IGF-1, T_4_ and T_3_ which are essential factors of bone longitudinal growth through enchondral ossification, as well as serum concentrations of osteocalcin, hydroxyproline and CTX-II which are biomarkers of bone and cartilage turn-over. Fasting glucose concentrations were measured at 3, 30, 90, 130, 180 and 210 days of age using an automated analyzer (Medisense Optium Xceed, Abbott, Illinois, USA). Plasma or serum was separated after centrifugation and stored at -20°C.

### Glucose metabolism testing in mares and foals

All metabolic tests were performed without analgesia or anesthesia.

#### Assessment of the daily effect of diet on plasma glucose and insulin

Jugular vein blood samples were collected each hour over a 10-hour period on December 17^th^ (median gestational day 212; range 171 to 223), starting at 8:30 AM and ending at 6:30 PM, for immediate measurement of plasma glucose concentrations using an automated analyzer. Plasma was separated after centrifugation and stored at -20°C.

#### Intravenous glucose tolerance test (IVGTT) in mares and foals

IVGTT were performed in mares in August (before dietary treatment, at median gestational age 96; range 53 to 107) and in February (after the start of the dietary treatment, at median gestational age 276; range 237 to 286), as well as in foals at median ages 3 and 130 days (range 125 to 138). The procedure and calculations were previously described [8].

Food was withheld from mares the evening before the procedure, whereas foals were muzzled to prevent suckling 4 h (at 3 days of age) and 6 h (at 130 days of age) before the procedure. Animals were intravenously infused with glucose (0.25 g/kg, 30% glucose) over 5 min in mares, 1 min in 3-day old foals and 2 min in 130-day old foals. Jugular vein blood samples were collected in EDTA tubes in mares at -5 min and 5, 7, 9, 12, 15, 30, 60, 90, 120, 150 and 180 min after the injection started, in 3-day old foals at -5 min and 1, 3, 5, 7, 9, 12, 15, 30 and 60 min after the injection started and in 130-day old foals at -5 min and 2, 3, 5, 7, 9, 12, 15, 30, 60 and 120 min after the injection started.

#### Hyperinsulinemic euglycemic clamp in foals

Hyperinsulinemic euglycemic clamps were performed in foals at median age 210 days (range 202 to 216). The procedure and calculations were previously described [8].

### Biochemical analyses

#### Plasma NEFA analysis

Plasma NEFA concentrations were measured in mares and foals in duplicate with an enzymatic-colorimetric method using a Cobas Mira-analyzer with a commercial kit (Roche, Mannheim, Germany) as previously described [8]. The minimum level of detection was 10 μmol/L. Intra- and inter-assay coefficients of variation were 2.7% and 4.5%, respectively.

#### Plasma leptin analysis

Plasma leptin concentrations were measured in mares and foals in duplicate with a homologous double-antibody RIA as previously described [8, 16]. The limit of detection was 1.0 ng/ml. The intra-assay coefficient of variation was less than 10%.

#### Plasma insulin analysis

Plasma insulin concentrations during glucose metabolism tests were measured in duplicate with a double antibody RIA as previously described [8, 16]. The minimum level of detection was 0.1 pg/mL. Intra- and inter-assay coefficients of variation were 7.2% and 5.8%, respectively.

#### Plasma IGF-1, T_4_ and T_3_ analyses

Plasma IGF-1, T_4_ and T_3_ concentrations were measured in foals in duplicate with commercial RIA kits (CISbio International, Gif-sur-Yvette, France) as previously described [8, 16]. The minimum levels of detection were 1.0 ng/mL, 2.5 ng/mL and 0.1 ng/mL, respectively for plasma IGF-1, T_4_ and T_3_. Intra- and inter-assay coefficients of variation were respectively 3.5% and 6.0% for plasma IGF-1, 4.7% and 8.0% for plasma T_4_ and 7.8% and 8.2% for plasma T_3_.

#### Serum osteocalcin, hydroxyproline and CTX-II analyses

Serum osteocalcin and CTX-II were assayed in foals in simple with commercial ELISA kits (Immunodiagnostic Systems, Paris, France) validated for use in the horse in the Frank Duncombe Departmental Laboratory. Photometric measurements were set at a wavelength of 450 nm. The minimum levels of detection were 0.5 ng/mL and 3.7 pg/mL, respectively for osteocalcin and CTX-II. Intra- and inter-assay coefficients of variation were respectively 4.0% and 1.8% for osteocalcin and 4.8% and 2.4% for CTX-II. Serum hydroxyproline concentrations were measured in foals in simple with a colorimetric assay as previously described [17, 18]. Photometric measurements were set at a wavelength of 558 nm.

### Radiographic evaluation of osteoarticular status in foals

Radiographic examination at median age 218 days (range 183 to 239) was performed according to a previously reported procedure [13]. Foals were sedated using romifidine (0.06 mg/kg intravenous of Sedivet, Boehringher Ingelheim, Belgium) alone or combined with butorphanol (0.02 mg/kg intravenous of Dolorex, Intervet/Schering Plough, Belgium) when needed. Two experimented examiners analyzed individually the digital radiographs, using the method of radiographic interpretation previously described [19]. Foals were classified as OC-positive according to the presence of one or more OC lesions identified by both examiners. OC was diagnosed according to the presence of lesions that were previously described [13, 20].

### Statistical analyses

Results are expressed as median [quartile 1—quartile 3] and presented as curves (median and interquartile range IQR) or boxplots (minimum to maximum). All data are showed in S1 to S8 Datasets. Statistical analyses were carried out using R software (www.r-project.org/, version 2.15.2).

In mares, repeated measures were divided in three periods (pregnancy before diet, pregnancy during dietary treatment and lactation) and were analyzed in each period by non-parametric ANOVA using the F1-LD-F1 model of the nparLD function [21], taking into account the diet and time. This analysis compares one factor (= whole-plot factor, in this case, group category) and one repeated factor (= sub-plot factor; in this case, diet category), applied only during a given time period, with repeated measurements for each subject (continuous parameter), as for a two factor ANOVA (one inter and one intra. The R input code is showed in S1 Text. If the dietary effect or diet:time interaction were significant, B and F mares were compared using the Mann-Whitney test at each time point. P-values from the Mann-Whitney tests were adjusted using the FDR (Benjamini-Hochberg) method.

In foals, repeated measures were analyzed using the F2-LD-F1 model of the nparLD function, taking into account the diet, sex and time [21]. This analysis compares two independent factors: group (category forage or barley) and sex (category male or female) with repeated measurements for each subject (continuous parameter), as for a three factor ANOVA (two inter and one intra). The R input code is showed in S1 Text. If the diet effect, diet:time interaction, sex effect or sex:time interaction were significant, B and F foals, as well as fillies and colts, were compared using the Mann-Whitney test at each time point. P-values from the Mann-Whitney tests were adjusted using the FDR (Benjamini-Hochberg) method. Non-repeated measures were analyzed using the Mann-Whitney test with no adjustment.

Effects were considered significant for p-values<0.05. For repeated measures, p-values in the text are those from the F1-LD-F1 and F2-LD-F1 tests, whereas asterisks in the figures indicate adjusted p-values<0.05 from the Mann-Whitney tests. For non-repeated measures, p-values in the text are those from the Mann-Whitney tests.

Relative risks and odds ratios related to osteochondrosis presence or absence were calculated using MedCalc software (http://www.medcalc.org/, v13.3.3) and analyzed using chi-squared tests. They were considered significant for p-values<0.05.

## Results

### Nutritional offer to mares

**Fig 1:**
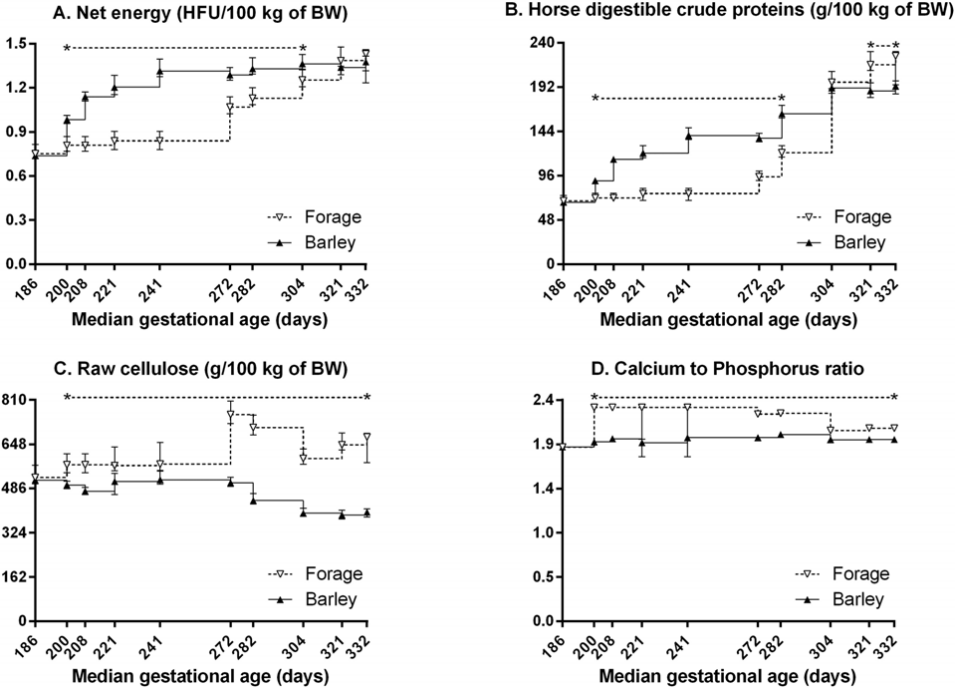
Daily nutritional offer (median and IQR) to broodmares in late pregnancy: net energy (A), horse digestible crude proteins (B), raw cellulose (C), and calcium to phosphorus ratio (D). HFU: horse feed units, BW: bodyweight. Values under the asterisks significantly differ between groups (Mann-Whitney test with FDR adjustment).

Fig 1 presents the nutritional offer to mares according to gestational age. It differed significantly between both diets for net energy (NE, expressed in horse feed units, p<0.001), horse digestible crude proteins (HDCP, p = 0.007), raw cellulose (RC, p<0.001) and calcium to phosphorus ratio (Ca/P, p<0.001), with significant diet:time interactions (p<0.001 for the four parameters).

The NE offer was significantly higher in B *versus* F mares from gestational days 200 to 321 and similar between both groups from gestational day 321 to parturition. Diet B provided up to 59.9% more NE than diet F from gestational days 241 to 272.

The HDCP offer was significantly higher in B *versus* F mares from gestational days 200 to 304. It was similar between both groups from gestational days 304 to 321, after which the HDCP supply was significantly lower in B *versus* F mares until parturition. Diet B provided up to 89.2% more HDCP than diet F from gestational days 241 and 272 and up to 14.6% less HDCP than diet F from gestational day 321 to parturition.

The RC and Ca/P offers were significantly lower in B *versus* F mares from gestational days 200 to parturition. Diet B provided up to 39.8% less RC from gestational day 321 to parturition.

### Effects of diets in mares

#### Pregnancy and parturition outcomes

Twenty-five pregnancies were obtained that were allocated to group F (n = 13) and group B (n = 12). All mares delivered spontaneously within the normal pregnancy length (range 331 to 356 days). One B foal with abnormal presentation caused dystocia in the mare and died. Pregnancy data relative to this mare were not discarded from further analysis. Twenty-four healthy foals were obtained (n = 13 in group F; n = 11 in group B): 7 fillies/6 colts in group F and 5 fillies/6 colts in group B. One F mare died and her colt was weaned at 150 days of age. Another F colt died around 150 days of age from septicemia. All other foals were weaned at 180 days of age.

#### Bodyweight and BCS in mares

**Fig 2:**
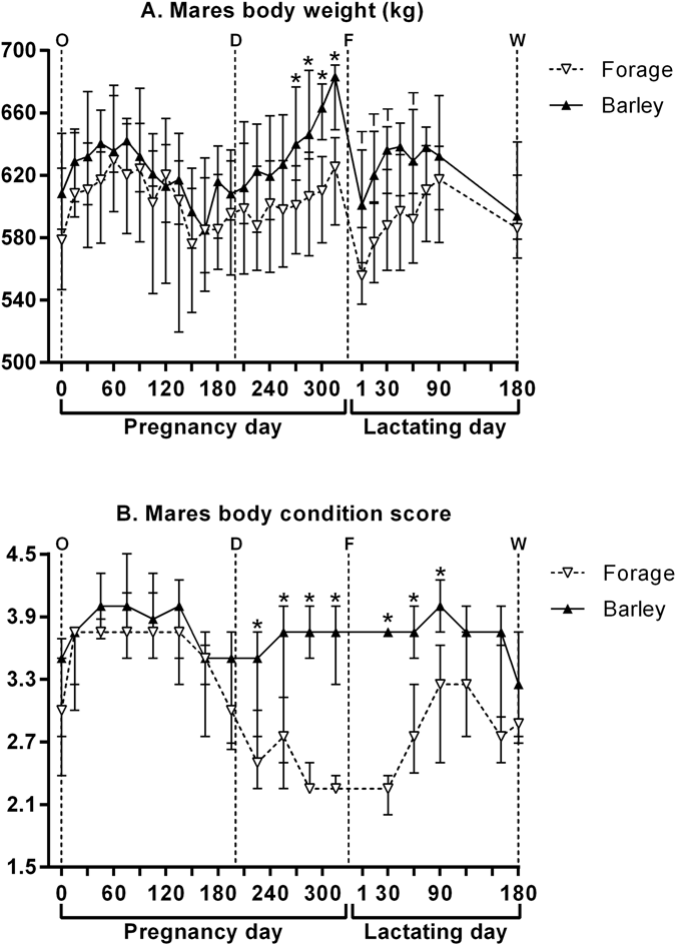
Mares’ bodyweight (A) and body condition score (B) (median and IQR) throughout pregnancy and lactation. O: ovulation, D: start of the diet, F: foaling, W: weaning. Values under the asterisks significantly differ between groups and those under letter “T” tend to differ between both groups (Mann-Whitney with FDR adjustment).

Fig 2 presents mares’ bodyweights and BCS. Statistical analyses of BCS were performed from the 2^nd^ month of pregnancy.

From gestational days 0 to 195, bodyweights were similar between both groups, but varied significantly with time (p<0.001). From gestational days 210 to 315, a significant dietary effect (p = 0.016) and time effect (p<0.001) were observed on bodyweights, with a significant diet:time interaction (p = 0.009). F mares were significantly lighter than B mares on gestational days 270 (-6.1%), 285 (-6.2%), 300 (-8.0%) and 315 (-8.5%). From suckling days 1 to 180, a significant time effect (p<0.001) and diet:time interaction (p = 0.034) were observed on bodyweights, whereas the dietary effect tended to be significant (p = 0.067). F mares tended to be lighter than B mares on suckling days 1, 15, 30 and 60.

From gestational days 45 to 195, BCS were similar between both groups, but varied significantly with time (p<0.001). From gestational days 225 to 315, no time effect was observed, but BCS were significantly affected by diet (p<0.001), with a significant diet:time interaction (p = 0.007). F mares had significantly lower BCS on gestational days 225, 255, 285 and 315. From suckling days 30 to 180, a significant dietary effect (p<0.001), time effect (p<0.001) and diet:time interaction (p<0.001) were observed. F mares had significantly lower BCS on suckling days 30, 60 and 90.

#### Glucose homeostasis in mares

**Fig 3:**
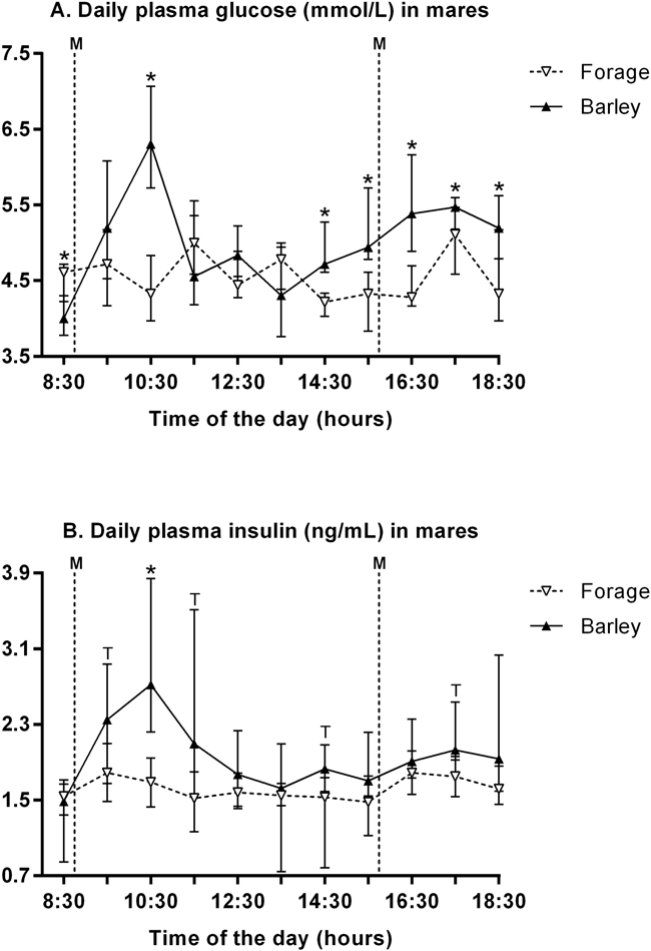
Mares’ plasma glucose (A) and insulin (B) concentrations (median and IQR) over a 10-hour monitoring period a fortnight after start of the diet. M: meals. Values under the asterisks significantly differ and those under letter “T” tend to differ between both groups (Mann-Whitney with FDR adjustment).

The effect of maternal diet and meal time on daily glucose and insulin concentrations was studied. Data are shown in Fig 3. Plasma glucose concentrations varied significantly with diet (p<0.001) and time of the day (p<0.001) with a significant diet:time interaction (p<0.001). B mares had significantly higher plasma glucose concentrations at 8:30 AM, 10:30 AM, 2:30 PM, 3:30 PM, 4:30 PM, 5:30 PM and 6:30 PM than F mares. Plasma insulin concentrations varied significantly with diet (p = 0.013) and time of the day (p<0.001) with a significant diet:time interaction (p = 0.015). B mares had significantly higher plasma insulin concentrations at 10:30 AM and tended to have higher concentrations at 9:30 AM, 11:30 AM, 2:30 PM and 5:30 PM.

**Fig 4:**
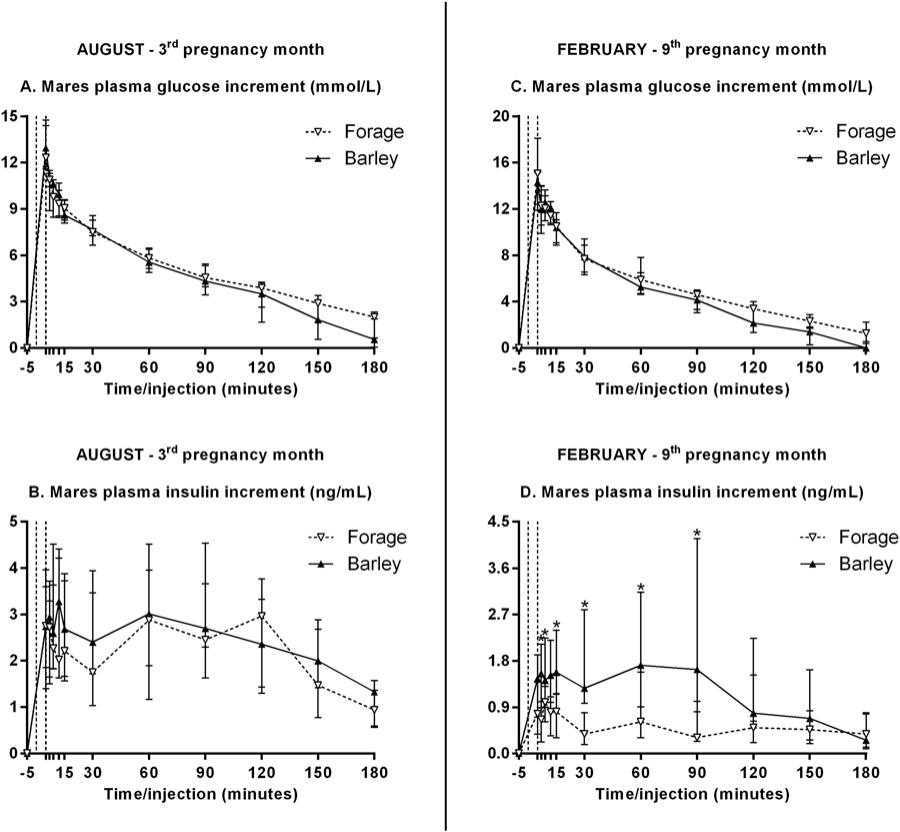
Mares’ plasma glucose and insulin increments (median and IQR) during IVGTT before (A, B) and after (C, D) start of the diet. The 5-min period between both dotted lines stands for the glucose injection time. Values under the asterisks significantly differ between both groups (Mann-Whitney with FDR adjustment).

IVGTT were performed before and after the dietary treatment started. Data are shown in Fig 4. No difference between groups was observed in August. Plasma glucose increments, AUC and maximal increment during IVGTT in February were unaffected by diet. Plasma insulin increments, however, were significantly higher in B *versus* F mares at 7, 12, 15, 30, 60 and 90 min after the injection started (p = 0.002 for the dietary effect). Plasma insulin AUC was significantly increased in B *versus* F mares (p = 0.009), and the maximal plasma insulin increment tended to be significantly higher in B *versus* F mares (p = 0.057).

#### Plasma NEFA and leptin in mares

**Fig 5:**
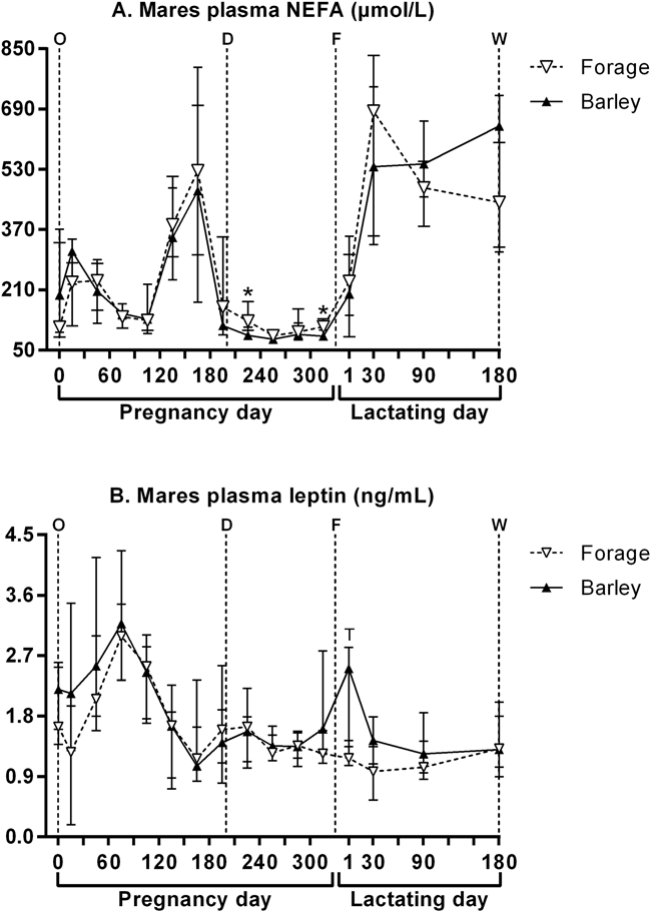
Mares’ plasma NEFA (A) and leptin (B) concentrations (median and IQR) throughout pregnancy and lactation. O: ovulation, D: start of the diet, F: foaling, W: weaning. Values under the asterisks significantly differ and those under letter “T” tend to differ between both groups (Mann-Whitney with FDR adjustment).

Fig 5 presents mares’ plasma NEFA and leptin concentrations. Statistical analyses of both parameters were performed from the 2^nd^ month of pregnancy.

From gestational days 45 to 195, plasma NEFA concentrations were similar between both groups but varied significantly with time (p<0.001). From gestational days 225 to 315, a significant dietary effect (p = 0.007) and time effect (p<0.001) were observed on plasma NEFA concentrations. F mares had higher plasma NEFA concentrations than B mares on gestational days 225 and 315. Throughout suckling, plasma NEFA concentrations varied significantly only with time (p<0.001).

From gestational days 45 to 195, plasma leptin concentrations were similar between both groups but varied significantly with time (p<0.001). From gestational days 225 to 315, no significant dietary or time effect was observed. Throughout suckling, plasma leptin concentrations varied significantly with time (p = 0.019) and the dietary effect and diet:time interaction tended to be significant (p = 0.089 and p = 0.090). F mares tended to have lower plasma leptin concentrations on suckling day 1.

### Effects of diets in foals

#### Body measurements in foals

Table 5 presents foals’ bodyweight, withers’ height and chest circumference at 1 and 180 days of age. All body measurements increased significantly with age (p<0.001) without any significant dietary or sex effect. Daily weight gain remained unchanged by diet or sex.

**Table 5:** Foals parameters in groups “forage” and “barley” (median [quartile 1-quartile 3]).

	Age (days)	Group “forage” (n = 13 or n = 12)	Group “barley” (n = 11)	Diet effect (F2-LD-F1 model)
**Bodyweight (kg)**	**1**	53.7 [50.0–59.7]	54.3 [50.3–58.3]	p = 0.72
	**180**	241.5 [224.1–266.5]	249.6 [243.5–252.4]	
**Withers’ height (cm)**	**1**	101.0 [100.0–102.5]	100.5 [99.3–103.0]	p = 0.35
	**180**	134.0 [130.5–136.0]	131.0 [130.0–133.5]	
**Chest circumference (cm)**	**1**	85.0 [85.0–90.0]	85.0 [82.3–86.3]	p = 0.94
	**180**	144.0 [139.3–146.8]	142.5 [140.0–143.5]	
**IGF-1 (ng/mL)**	**0**	164.6 [147.4–194.2]	189.9 [165.2–200.7]	p = 0.53
	**180**	272.7 [223.8–382.8]	335.6 [287.1–362.3]	
**T**_**4**_ **(ng/mL)**	**0**	408.4 [328.6–427.1]	370.7 [352.9–423.3]	p = 0.25
	**180**	26.4 [21.0–29.2]	22.4 [18.3–24.6]	
**T**_**3**_ **(ng/mL)**	**0**	5.7 [4.2–6.5]	5.4 [3.9–8.5]	p = 0.53
	**180**	0.36 [0.31–0.53]	0.36 [0.31–0.44]	
**NEFA (μmol/L)**	**3**	787.0 [612.0–889.0]	679.0 [452.5–822.5]	p = 0.77
	**180**	647.5 [417.5–924.5]	660.0 [426.0–912.5]	
**Osteocalcin (ng/mL)**	**3**	68.9 [40.8–92.0]^T^	107.6 [56.2–165.2]	**p = 0.054**
	**130**	16.2 [13.3–21.6]	15.0 [9.9–22.5]	
**Hydroxyproline (mg/L)**	**3**	18.5 [14.5–21.3]	16.8 [15.6–18.2]	p = 0.96
	**130**	8.3 [7.9–8.8]	8.8 [8.6–8.8]	
**CTX-II (ng/mL)**	**3**	2.5 [1.9–2.6]	2.7 [2.4–3.8]	p = 0.11
	**130**	1.1 [1.0–1.1]	1.1 [1.1–1.2]	
	**Age (days)**	**Group “forage” (n = 13 or n = 12)**	**Group “barley” (n = 11)**	**Diet effect (Mann-Whitney test)**
**Leptin (ng/mL)**	**180**	1.3 [1.2–1.4]	1.3 [1.3–1.4]	p = 0.70
**Glucose AUC (mmol.min/L)**	**3**	114.8 [106.6–133.7]	99.5 [77.1–120.4]	**p = 0.087**
	**130**	411.0 [338.9–498.1]	409.6 [382.5–448.4]	p = 0.75
**Insulin AUC (ng.min/L)**	**3**	28.6 [20.0–37.4]	23.8 [17.8–29.9]	p = 0.27
	**130**	30.2 [17.1–46.9]	33.7 [22.0–42.3]	p = 0.79
**Glucose metabolism rate (mmol/kg/min)**	**200**	0.016 [0.014–0.022]	0.017 [0.016–0.021]	p = 0.54

P-values are given for the diet effect on each parameter. For repeated measures, p-values are those obtained using the F2-LD-F1 model. For non-repeated measures, p-values are those obtained using the Mann-Whitney test.

#### Glucose homeostasis in foals

Fasting glycemia decreased significantly as foals became older (p<0.001), reached a nadir at 90 days of age and remained stable thereafter. Fasting glycemia was unaffected by diet or sex.

Table 5 presents data from glucose metabolism testing. During IVGTT at 3 days of age, plasma glucose concentrations peaked 3 min after the start of the glucose injection. Plasma glucose increments were significantly affected by diet (p = 0.012), tended to be higher 3 min post-injection and were significantly higher 5 min post-injection in F *versus* B foals. Plasma glucose AUC tended to be higher in F *versus* B foals (p = 0.087) and maximal increments were unaffected by diet. Plasma insulin concentrations peaked 5 min after the glucose injection started. Plasma insulin increments, AUC and maximal increments were unaffected by diet. During IVGTT at 130 days of age, plasma glucose and insulin increments were not affected by diet anymore. No sex effect was observed at both ages.

No dietary effect was observed on glucose metabolism rates during hyperinsulinemic euglycemic clamps at 210 days of age. Fillies, however, had a significantly higher glucose metabolism rate than colts (p = 0.042).

#### Plasma NEFA and leptin in foals

Table 5 presents foals’ plasma NEFA concentrations at 3 and 180 days and plasma leptin concentrations at 180 days. No effect of age, diet or sex was observed on both parameters.

#### Plasma IGF-1, T_4_ and T_3_ in foals

Table 5 presents plasma IGF-1, T_4_ and T_3_ concentrations at 0 and 180 days of age. Plasma IGF-1 significantly increased whereas T_4_ and T_3_ concentrations significantly decreased as foals became older (p<0.001 for the three parameters), without any significant dietary effect. Plasma IGF-1 concentrations were subject to a significant sex effect (p = 0.008), with fillies having significantly lower plasma concentrations than colts at birth, 130 and 180 days of age. Plasma T_4_ and T_3_ concentrations were unaffected by sex.

#### Serum osteocalcin, hydroxyproline and CTX-II

Table 5 presents serum osteocalcin, hydroxyproline and CTX-II concentrations at 3 and 130 days. All three parameters decreased significantly as foals became older (p<0.001 for all three parameters). Only serum osteocalcin concentrations tended to be affected by diet (p = 0.054) with a diet:time interaction (p = 0.055). F foals tended to have lower serum osteocalcin concentrations than B foals at 3 days of age. No sex effect was found on any of the three parameters.

#### Osteochondrosis lesions

Seven foals were osteochondrosis-positive at 218 days of age: 2 fillies in group F (16.7%) *versus* 5 foals (4 fillies and 1 colt) in group B (45.5%). Neither relative risks nor odds ratios differed between both groups (Table 6). Both affected foals of group F presented 2 osteochondrosis lesions, localized in the right hock and left stifle for one foal and in both hocks for the other foal. The five affected foals of group B presented only one osteochondrosis lesion, localized in one or the other of the hocks for 2 of them, localized in one or the other of the stifles for 2 other foals and in the left front fetlock for the last one.

**Table 6:** Foals’ relative risk and odds ratio of osteochondrosis in groups “forage” and “barley” at 218 days of age.

	Group “forage” (n = 12)	Group “barley” (n = 11)	Z statistic	Significancelevel
**Number of unaffected foals**	10	6		
**Number of osteochondrosis positive foals**	2	5		
**Relative risk**	0.3667	2.7273	1.384	p = 0.1665
**95% confidence interval**	0.08852–1.5187	0.6584–11.2964		
**Odds ratio**	0.2400	4.1667	1.452	p = 0.1466
**95% confidence interval**	0.0394–1.6486	0.6066–28.6219		

## Discussion

Cracked barley provided additional net energy and digestible proteins to mares from the 7^th^ month of pregnancy. Barley containing meals induced a sharp peak of both glycemia and insulinemia in the morning, followed by a second rise of glycemia in the afternoon associated with a slight increase of insulinemia. Barley enabled mares to maintain optimal body condition throughout pregnancy and lactation and to gain bodyweight until parturition. On the opposite, mares fed forage dramatically lost body condition until return to pasture and gained less bodyweight throughout pregnancy. They demonstrated energy deficit as shown by transient higher plasma NEFA concentrations during dietary treatment. In the 9^th^ month of pregnancy, insulin secretion in response to glucose injection was markedly lowered in mares fed forage, with unaffected glucose clearance.

No difference was found in pregnancy length, foals’ measurements or endocrine factors of growth from birth until weaning. F foals, however, tended to be more tolerant to glucose at 3 days of age, as demonstrated by amplified glucose response and slowed-down glucose clearance after injection, without any variation in insulin response. F foals also had lower osteocalcin concentrations at the same age. Those alterations were corrected at the time of weaning. Preliminary data on osteoarticular status shortly after weaning showed increased incidence of osteochondrosis lesions in foals born to dams fed barley but this was not significant.

### Nutritional offer to mares

B mares received more net energy (up to 59.9% more) and digestible proteins (up to 89.2% more) than F mares between the 7^th^ and 10^th^ month of pregnancy. Energy supply was similar between both groups thereafter, whereas F mares received more proteins than B mares. Hay batches given from the 10^th^ month of pregnancy differed from that used from the 7^th^ to 10^th^ month of pregnancy by their nutritional properties, with higher energy, nitrogen, phosphorus and calcium contents. Energy provided by each diet differed by their amounts, but also by their nature: forage provides mainly energy derived from volatile fatty acids, whereas barley provides energy derived from simple carbohydrates. Nitrogen concentrations in both diets did not exceed 100% of current INRA recommendations for broodmares [14]. Besides, the calcium to phosphorus ratio remained in a range from 1 to 3, as recommended to avoid calcium absorption defects and bone demineralization due to phosphorus excess, as well as magnesium and trace elements (manganese, zinc, iron, copper and iodine) absorption defects due to calcium excess [14]. On the opposite, B mares received lower amounts of fiber (up to 39.8% less) *i.e*., less than 30% fiber whereas F mares received between 28% and 35% fiber. Fiber excess (>30%), however, is susceptible to limit digestibility of other compounds of the diet, as well as the feed intake necessary to cover nutritional requirements [14].

### Effects of diets in mares

B mares maintained BCS higher than 3.5 until parturition which is considered optimal for future lactation. On the opposite, F mares’ BCS fell below 2.5 until parturition which is considered insufficient [15]. F mares’ BCS did not start to increase before the 2^nd^ suckling month. This could have altered milk composition since reforming body stores prevails over lactation in mares. Indeed, in thin mares, close to 30% of the energy intake enables stores deposit, whereas in fat mares, most energy is used for milk lipids synthesis [22]. Such a loss of body condition and such lower bodyweights were previously reported in Quarter Horse mares conducted in pasture (estimated to receive 100% of NRC digestible energy recommendations) *versus* mares conducted in pasture and supplemented with grain mix (120% of NRC digestible energy recommendations) in the last third of pregnancy. Light breed mares receiving low *versus* high starch isocaloric and isonitrogenous diets from the 7^th^ month of pregnancy also lost body condition and presented lower bodyweights, even if to a lesser extent [12].

F mares presented a transient increase in plasma NEFA concentrations at the start and at the end of the dietary treatment compared to B mares. This suggests increased lipids mobilization in lean mares, as previously described in non-pregnant light breed mares in which digestible energy restriction increased by 2.5 plasma NEFA concentrations [23]. Plasma leptin concentrations, which are related to fat deposit and BCS in mares [24], were mainly unchanged between both diets. Only a tendency for lower plasma leptin concentrations was found in F mares on the 1^st^ suckling day, although a decrease could also be expected before parturition as shown in mares conducted in pasture only [25] or fed a moderate energy diet [26]. Decreased plasma leptin concentrations are consistent with the lower BCS observed in our mares, but variations are so weak that they moderate potential increased lipids mobilization suggested above.

Plasma glucose and insulin concentrations in B mares peaked within 2 h after the 1^st^ meal and only slightly increased after the 2^nd^ meal, as was found in non-pregnant mares receiving various amounts of supplements of varying composition (mainly cracked corn, cottonseed hulls, corn gluten meal and molasses) [23]. Daily plasma glucose and insulin concentrations in response to feeding or fasting were in the same range as reported in non-pregnant mares [23], young geldings [27] and adult mares and stallions [28]. On the other side, energy and nitrogen restriction did not affect glucose clearance rate, but decreased insulin secretion during IVGTT in F mares in the 9^th^ month of pregnancy. This is consistent with reports of unaffected glucose clearance rate but reduced insulin secretion during IVGTT in non-pregnant mares fed half energy and half protein requirements (both groups receiving concentrates). Frequently-sampled intravenous glucose tolerance test (FSIGT) in pregnant mares fed high *versus* low energy diets in the last trimester, however, did not evidence any alteration in insulin sensitivity, glucose effectiveness or beta-cell responsiveness, nor in late pregnancy neither later in lactation [29]. But the authors repeated the experiment in order to increase variations in body condition between both groups (high BCS in the high energy fed group *versus* low BCS in the low energy fed group), so that they induced lower insulin sensitivity and lower acute insulin response to glucose in mares fed the low energy diet [30]. This diminished responsiveness of beta-cells is consistent with our results. This is surprising since insulin sensitivity is known to decline throughout pregnancy [31]. We could have expected amplified beta-cell secretion to compensate for the lower insulin-mediated glucose uptake. Such an inadequate beta-pancreatic function, together with severe insulin resistance reflects gestational diabetes mellitus in thin women [32], quite similarly to our low-BCS mares fed forage only.

### Effects of diets in foals

The diets that we used elicited different glucose and insulin responses in mares. Thus, fetal development could have been affected by barley supplementation. Pregnancy length and foals’ body measurements at birth were similar between both groups. This is consistent with previous work inducing dietary metabolic variations in pregnant mares [11, 12, 25, 33]. As discussed above, body condition of mares could have affected immunoglobulins concentrations in colostrum [33] or milk composition [22]. Milk data are not available at this time. Foals’ growth, however, is used as an indirect marker of milk yield. Here, none of the foals’ body measurements was affected by the dams’ diet until weaning. These data are consistent with similar leptin concentrations at weaning, indicating similar fat deposit in both groups at this age. We were not able to measure foals’ plasma leptin concentrations earlier in the suckling period but foals born to dams fed either a high or low energy diet were shown to have similar plasma leptin concentrations in the first two weeks [26]. In the same way, all foals had similar lipid mobilization as shown by similar plasma NEFA concentrations until weaning.

Here, fasting glucose concentrations from birth to weaning were independent from the mares’ diet. Energy and nitrogen restriction in late pregnancy, however, slightly affected glucose tolerance of F foals in the immediate post-natal period. F foals had similar insulin secretion to B foals but slowed-down glucose clearance rate. This coincides with lower insulin sensitivity and glucose effectiveness observed during FSIGT in 2-week old foals born to dams fed a low energy diet [29]. We found that all effects on glucose and insulin homeostasis had vanished in F foals at weaning and again, this is consistent with data from 4-month old foals born to dams fed a low energy diet [29]. Concomitantly, F foals tended to have lower serum osteocalcin concentrations. Osteocalcin is a hormone released by osteoblasts which stimulates beta-cell proliferation and expression of insulin-encoding genes in beta-cells [34]. Its action on adipocytes also increases adiponectine release, which improves tissues sensitivity to insulin by enhancing glucose uptake by cells [35]. This could be an explanation for a tendency for higher glucose tolerance in F foals.

It is established that cereals-rich rations of foals increase their predisposition to osteochondrosis [36–40], because they enhance postprandial glycemia and evoke a peak of insulinemia [41]. Insulin is involved in cartilage metabolism through the Growth Hormone, IGF-I [42] and thyroid hormones [43], reducing chondrocytes maturation and bone formation. To date, only one epidemiological study demonstrated an adverse effect of concentrates in the ration of the pregnant mare on the development of osteochondrosis in foals [13]. Here, radiographic examination showed that F foals were less affected by osteochondrosis than B foals in the immediate post-weaning period, even if not significant. These could be related to the lower serum osteocalcin concentrations observed in B foals earlier and would be consistent with previous report in foals with severe radiographic findings [18]. This is in favor of a deleterious impact of feeding pregnant mares with concentrates in late pregnancy on osteoarticular status in foals. But osteochondrosis is a dynamic pathology and most lesions are susceptible to have evolved at one year of age. Further examination is needed to confirm this observation.

### Conclusion

In conclusion, our management of broodmares feeding elicits two different metabolic responses as regards energy balance and glucose homeostasis. Mares fed forage seem to develop a syndrome closed to gestational diabetes mellitus in women. This is worrying since it has been demonstrated to persist in the following pregnancy [44]. Few effects observed on foals’ metabolism had disappeared at weaning, but other factors could have been affected and any events in later life could reveal new programming aspects by the plane of nutrition of pregnant mares.

## Supporting Information

S1 Dataset Mares’ features and body measurements. (XLS)

S2 Dataset Mares’ daily plasma glucose and insulin concentrations. (XLS)

S3 Dataset Mares’ plasma glucose and insulin concentrations during IVGTT in August and February. (XLS)

S4 Dataset Mares’ blood parameters. (XLS)

S5 Dataset Foals’ body measurements. (XLS)

S6 Dataset Foals’ plasma glucose and insulin concentrations during metabolic tests at 3, 130 and 210 days of age. (XLS)

S7 Dataset Foals’ blood parameters. (XLS)

S8 Dataset Foals’ osteochondritic status. (XLS)

S1 Text R input code for F1-LD-F1 and F2-LD-F1 tests. (PDF)
