# Correction: Effects of Moderate Amounts of Barley in Late Pregnancy on Growth, Glucose Metabolism and Osteoarticular Status of Pre-Weaning Horses

**DOI:** 10.1371/journal.pone.0167604

**Published:** 2016-12-02

**Authors:** Pauline Peugnet, Morgane Robles, Luis Mendoza, Laurence Wimel, Cédric Dubois, Michèle Dahirel, Daniel Guillaume, Sylvaine Camous, Valérie Berthelot, Marie-Pierre Toquet, Eric Richard, Charlotte Sandersen, Stéphane Chaffaux, Jean-Philippe Lejeune, Anne Tarrade, Didier Serteyn, Pascale Chavatte-Palmer

The term “nutritional offer” appears incorrectly throughout the paper. The correct term should be “nutritional intake.”

Fig 1 and its caption appear incorrectly in the published article. Please see the correct Fig 1 and its caption below.

**Fig 1:**
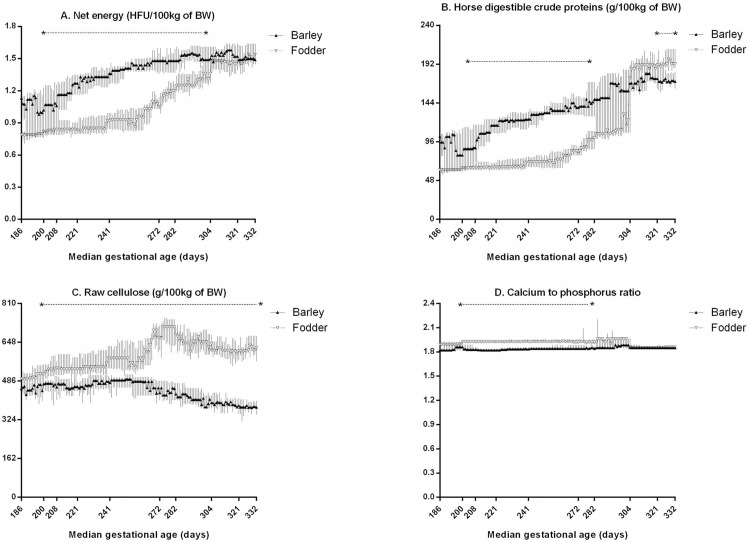
**Daily nutritional intake (median and IQR) to broodmares in late pregnancy: net energy (A), horse digestible crude proteins (B), raw cellulose (C), and calcium to phosphorus ratio (D).** HFU: horse feed units, BW: bodyweight. Values under the asterisks significantly differ between groups (Mann-Whitney test with FDR adjustment).

There are errors in Table 2 of the published article. Please see the correct Table 2 and its caption below.

**Table 2:** Quality of feedstuff given to broodmares of groups “forage” and “barley” from November (median gestational day 186) to parturition.

	Chemical composition (per kg of dry matter)			Mineral composition (per kg of dry matter)	
	Net energy (Horse feed units)	Horse digestible crude proteins (g)	Raw cellulose (g)	Calcium (g)	Phosphorus (g)
**Homemade mix**	1.2	127	52.7	12.1	5.8
**Haylage**	0.9	88	249.0	7.7	3.8
**Hay H1**	0.5	30	372.0	3.7	2.1
**Hay H2**	0.6	88	30.8	6.6	3.8
**Excel Prima S**^**®**^	NA	NA	NA	375	62.5
