# Supplementary material for: Effects of Moderate Amounts of Barley in Late Pregnancy on Growth, Glucose Metabolism and Osteoarticular Status of Pre-Weaning Horses
Source: PLoS One. 2015 Apr 13;10(4):e0122596. doi: 10.1371/journal.pone.0122596 (PMC4395399; doi:10.1371/journal.pone.0122596)
Supplement: S1 Text — (PDF) [file pone.0122596.s009.pdf]

R input code for the F1-LD-F1 test:

```
library(nparLD)

var<-tab2[,"Measure"]
time<-tab2[,"Time"]
group<-tab2[,"Group"]
subject<-tab2[,"Mare"]

res.Dataset<-
f1.ld.f1(var,time,group,subject,time.name="Time",group.name="Group",description=FALSE)
res.Dataset$ANOVA.test
```

R input code for the F2-LD-F1 test:

```
library(nparLD)

var<-Dataset[,"Measure"]
time<-Dataset[,"Time"]
group1<-Dataset[,"Group"]
group2<-Dataset[,"Sex"]
subject<-Dataset[,"Foal"]

res.Dataset<-
f2.ld.f1(var,time,group1,group2,subject,time.name="Time",group1.name="Group",group2.name="Sex",
description=FALSE)
res.Dataset$ANOVA.test
```
